# Generic parameters of first-order kinetics accurately describe soil organic matter decay in bare fallow soils over a wide edaphic and climatic range

**DOI:** 10.1038/s41598-019-55058-1

**Published:** 2019-12-30

**Authors:** Lorenzo Menichetti, Göran I. Ågren, Pierre Barré, Fernando Moyano, Thomas Kätterer

**Affiliations:** 1 Department of Ecology, Swedish University of Agricultural Sciences (SLU), Box 7044, 75007 Uppsala, Sweden; 2 Laboratoire de Geólogie de l’ENS, PSL Research University – CNRS UMR8538, 75005 Paris, France; 3 Georg-August Universität Göttingen, Büsgenweg 2, 37077 Göttingen, Germany

**Keywords:** Carbon cycle, Carbon cycle, Carbon cycle, Carbon cycle, Geochemistry

## Abstract

The conventional soil organic matter (SOM) decay paradigm considers the intrinsic quality of SOM as the dominant decay limitation with the result that it is modelled using simple first-order decay kinetics. This view and modelling approach is often criticized for being too simplistic and unreliable for predictive purposes. It is still under debate if first-order models can correctly capture the variability in temporal SOM decay observed between different agroecosystems and climates. To address this question, we calibrated a first-order model (Q) on six long-term bare fallow field experiments across Europe. Following conventional SOM decay theory, we assumed that parameters directly describing SOC decay (rate of SOM quality change and decomposer metabolism) are thermodynamically constrained and therefore valid for all sites. Initial litter input quality and edaphic interactions (both local by definition) and microbial efficiency (possibly affected by nutrient stoichiometry) were instead considered site-specific. Initial litter input quality explained most observed kinetics variability, and the model predicted a convergence toward a common kinetics over time. Site-specific variables played no detectable role. The decay of decades-old SOM seemed mostly influenced by OM chemistry and was well described by first order kinetics and a single set of general kinetics parameters.

## Introduction

Soil organic carbon (SOC) is the largest Earth surface C reservoir and one of the biggest hopes for climate change mitigation through C sequestration^1^. However, the estimate of how much C can be globally stored in soil is still lacking precision^2–4^. An important characteristic of SOC, which also defines the amount of C that a certain soil can store, is its *persistence*, a term we here use to refer to its mean lifetime and that is dependent on what we define here as SOC *quality*. At the macro or bulk level, the mean SOC lifetime will depend on the rate of SOC loss from the system. This has been historically considered to be mainly a consequence of substrate chemistry^5^ and generic kinetic parameters, but in recent years it has been proposed that such persistence is more a function of local ecosystem interactions^6^ and in particular of more complex metabolic interactions^7^.

The background question for the debate is how we should define the SOC models that are used for future projections of soil C stocks and whose results are used for guiding global policies, therefore with potentially large economic and social impacts. The decay of SOC is described in most current models with first order kinetics (where the rate of loss is defined by SOC stocks multiplied by a kinetic term, so losses are in absolute terms proportional only to SOC stocks), with additional modifiers to represent the effects of other factors. These can be considered time-varying, such as climate drivers, or constant, such as texture. Functions to represent temperature and moisture effects on SOC decay are present in basically all models^8^, with the general temperature response being well characterized^9^, while more uncertainty is often associated with the moisture response^10^. These rate modifiers are generally taken to be fully independent of the amount of substrate^11^. While *time-invariant* first order kinetics and *time-variant* climatic effects have been long represented in current SOC models, the importance of simulating metabolic interactions is still under active debate^12,13^. In recent years, several authors in the scientific community have strongly promoted the inclusion of higher order kinetics^7,14^, where the rate of SOC loss is a function of SOC multiplied by a variable kinetic term, itself a function of SOC, of microbial biomass, or of other factors. This comes from the perceived need to represent the interactions between microbial biomass and nutrient pools to capture SOC dynamics realistically. The explicit representation of microbial metabolism in SOC models could pay back in terms of prediction accuracy^7^. Still, it also implies costs in terms of model development and the need to estimate additional (and difficult to measure) parameters^15,16^. There are as well costs in terms of mathematical tractability, in particular the possibility of finding analytical solutions^17^. On the other hand, conventional first-order models can still represent a local (although constant over time) variation of metabolic parameters by varying the decay rates as a function of other factors (e.g. texture or nutrients) or with a discrete classification of model parameters related to ecosystem or soil type. This brings us to the key question of this work: how far can we push first-order SOC models in order to describe the variability of C dynamics in agroecosystems? Can first-order kinetics be enough to describe SOC persistence even over a wide geographical range?

To answer these questions we need to rely on long-term field trials, controlled outdoor experiments run for multiple decades. This adds several uncontrolled variables to account for compared to short-term laboratory setups, one being the variable input of C over the experimental duration. Such inputs constantly modify SOC quality and can cause nonlinear effects on SOC kinetics, in particular under significant priming^18^, which if present would be more problematic to represent for a first-order model.

To study the decay of SOC in the field without interference from fresh C input we can use long-term bare fallow (LTBF) experiments. Such multi-decadal experiments comprise soils that are weeded regularly to keep them free from vegetation, thus attaining long term C input values very close to zero. These setups are in general quite rare, but six sites exist in five European countries (Denmark, France, Russia, United Kingdom and Sweden)^19^ (Table 1). These experiments encompass a relatively broad range of soils, land use history and climate conditions and are therefore the ideal test ground for answering our questions. Some of these sites have been utilized in previous model development, but these efforts have been restricted to one single site at a time^20,21^. In this study we utilized the Q model^5^ (a first-order kinetics SOC model) to describe the evolution of SOC quantity and quality observed in all the LTBFs. The Q model defines quality of SOC by assuming that each atom of C has a quality value proportional to the time needed for the microbes to decompose it, and simulates the evolution of the average SOC quality $\bar{q}$ over time by a dispersion function $D=(\overline{\bar{q},\bar{q}^{\prime} )}$^22^. Compared with first-order compartmental SOC models, where the proportion of C in different compartments defines SOC quality, Q assumes SOC quality to be continuous or comprised of an indefinite number of pools.

**Table 1:** The sites considered. Unless specified otherwise, data are from^19^.

Country	Askov 1	Askov 2	Grignon	Kursk	Rothamsted	Ultuna	Versailles
	Denmark	Denmark	France	Russia	United Kingdom	Sweden	France
Longitude	55°28 N	55°28 N	48°51 N	51°73 N	51°82 N	59°49 N	48°48 N
Latitude	9°07E	9°07E	1°55E	36°19E	0°35E	17°38E	2°08E
Mean annual Temperature (°C)	7.8	7.8	10.7	5.4	9.5	5.5	10.7
Annual precipitation (mm)	862	862	649	574	712	533	628
Plot size (m.m-1)	11.7 × 9.4	11.7 × 9.4	3.2 × 3.2	10 × 10	7 × 12.5	2 × 2	2 × 2.5
Starting date	1956	1956	1959	1965	1959	1956	1928
Last sampling date	1985	1985	2007	2001	2008	2007	2008
Soil type (FAO)	Orthic Luvisol	Orthic Luvisol	Luvisol	Haplic Chernozem	Chromic Luvisol	Eutric Cambisol	Luvisol
Sampling soil depth (cm)	20	20	25	25	23	20	25
N of replicates	4	4	6	1	4	4	6
Clay (%)	7	7	30	30	25	36	17
Silt (%)	11	11	54	65	62	41	57
Sand (%)	82	82	16	5	13	23	26
pH	5.5–6.5	5.5–6.5	8–8.3	6.5	6.3	NA	6.4
Start bulk density (kg.dm-3)	1.50	1.50	1.20	1.13	0.94	1.44	1.30
Final bulk density (kg.dm-3)	1.50	1.50	1.21	1.13	1.43	1.43	1.44
Initial C stock (Mg C ha-1)	52.1 ± 5.9	47.7 ± 1.5	41.6 ± 2.7	100.29 ± N.A^68^.	71.7 ± 2.0	42.5 ± 2.4	65.5 ± 4.3
Final stock (Mg C ha-1)	36.4 ± 2.5	33.0 ± 2.5	24.5 ± 1.5	79.38 ± N.A^68^.	28.6 ± 3.1	26.9 ± 0.6	22.7 ± 3.3
Initial C/N ratio	10.27^37^	10.27^37^	8.44^37^	13.5^45^^†^	N.A.	9.35^37^	10.15^37^

^†^Data from a site close to the LTBF.

First-order decomposition parameters in the model were assumed general and valid at all the sites. These are (following the naming convention of the original paper by Bosatta and Ågren^22^) the term describing the shift in quality, *η*_11_ (that simplifies the quality dispersion function), and the term defining the decomposer growth rate, *μ*_0_.

Initial conditions (initial average litter input quality ${\bar{q}}_{o}$, which conceptually corresponds to the initialization of pools in a compartmental model) and edaphic kinetic modifiers (*β*, an exponential shape parameter that refers to how rapidly the decomposer growth rate changes with quality, which can be influenced by soil texture) were instead assumed to be variable between locations. Initial average litter quality ${\bar{q}}_{o}$ refers in Q to the quality of the inputs before the start of each LTBF experiment, and assuming steady state conditions then, it is proportional to initial SOM quality in the field experiments. In order to assess if also metabolic processes are needed to explain local variability, we also calibrated the efficiency of the soil decomposers (*e*_0_) as a site-specific local parameter, considered here independent from SOC variation. We calibrated the model within a Monte Carlo Markov chain (with a Metropolis-Hastings sampler) and a set of prior distributions for the parameters taken mainly from the literature, and we express therefore posterior parameter values as probability distributions (derived from the prior distributions and the data).

With this setup, we could test if a first-order kinetics SOC model is able to represent the decadal decay of SOC over a wide range of initial conditions, soils and climates, and identify which parameters (representing different processes) are most relevant for describing local variability of SOC decay kinetics.

## Results and Discussion

### Model parameterization results

**Figure 1:**
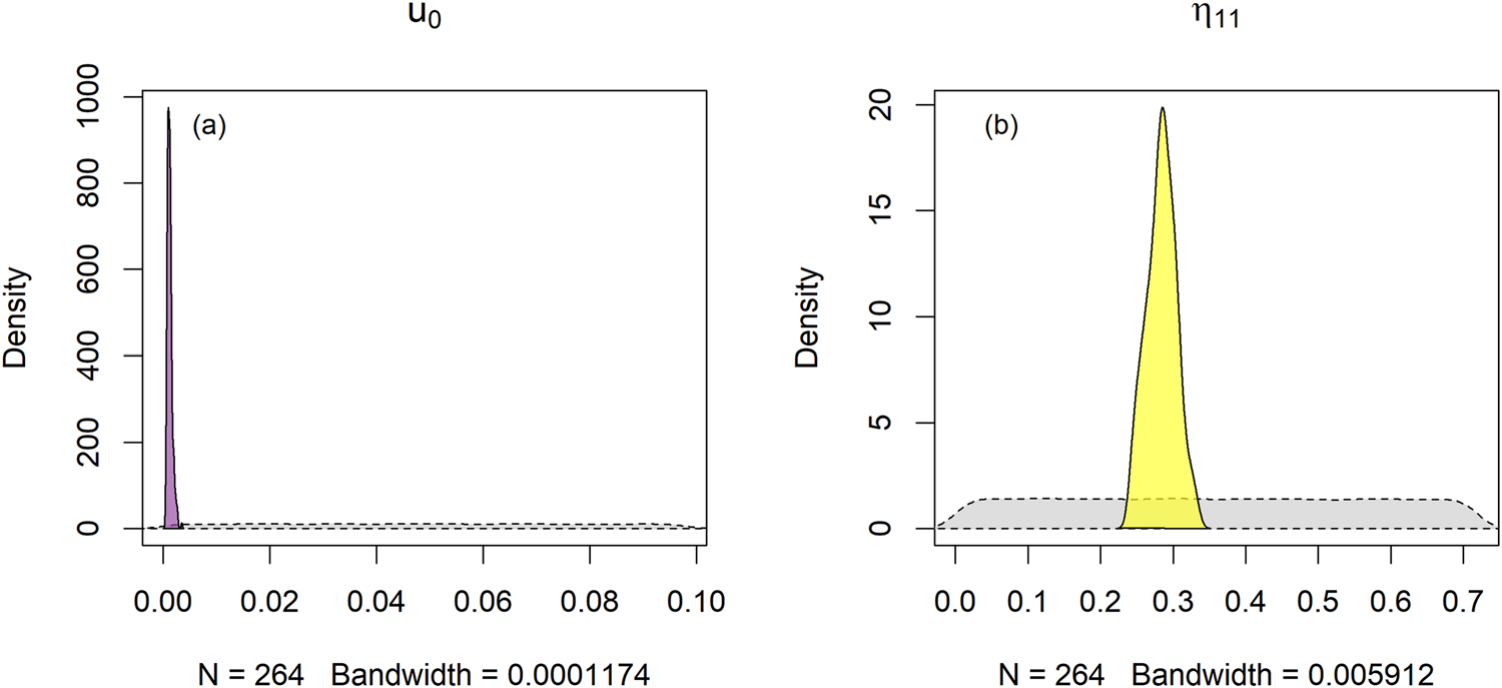
The generic parameters u_0_ and η_11_ (grey dashed area represent the priors). N indicates the number of the subsample utilized to calculate the density (a random sample of the MCMC), while the bandwidth is a parameter that roughly describes the granularity of the kernel density smoothing and is optimized by the algorithm used for the estimate (R function “density”^62^) with default values.

**Figure 2:**
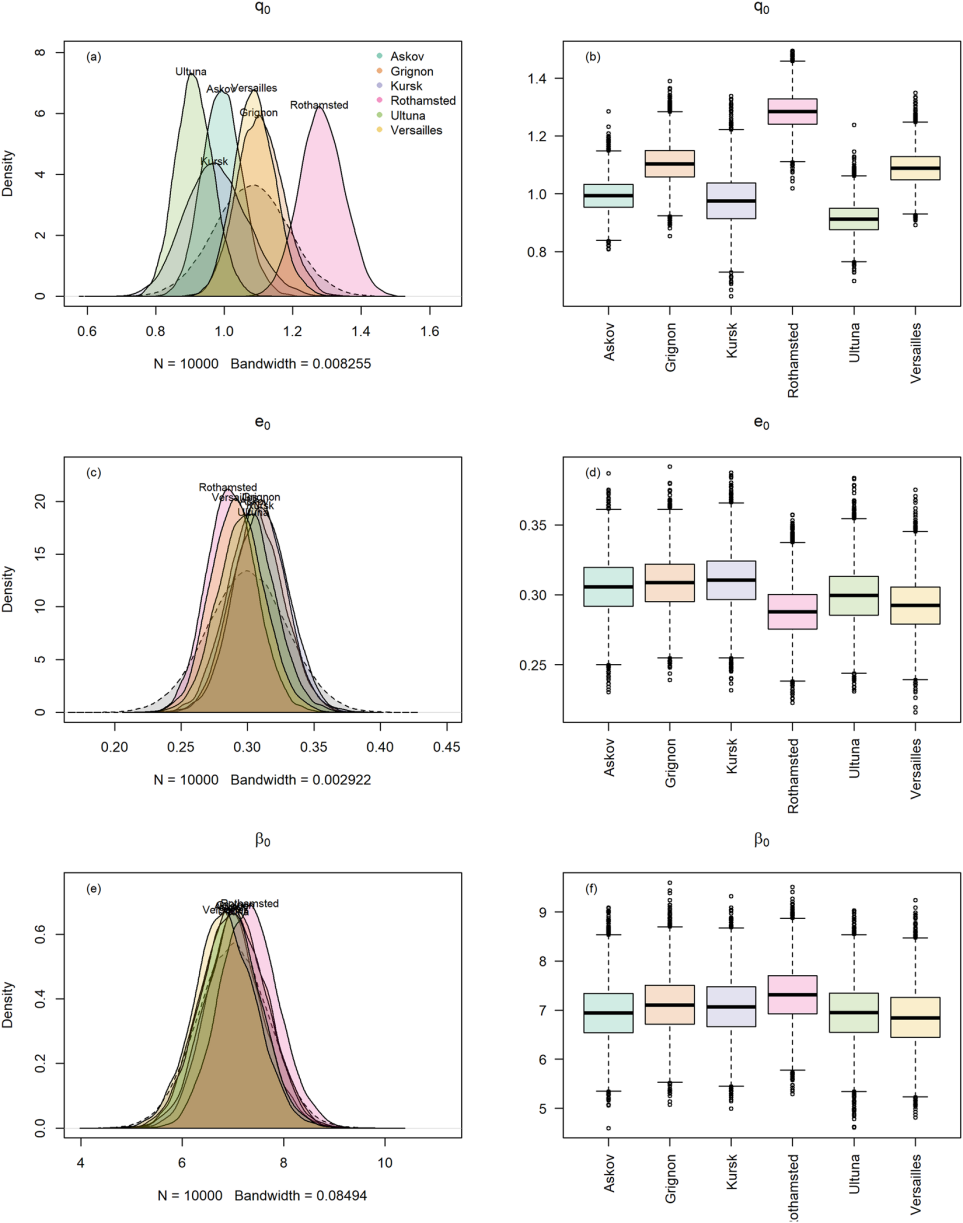
The local model parameters q_0_, e_0_, and β_0_ (grey dashed area represents the priors). N indicates the number of the subsample utilized to calculate the density (a random sample of the MCMC), while the bandwidth is a parameter that roughly describes the granularity of the kernel density smoothing and is optimized by the algorithm used for the estimate (R function “density”^62^) with default values. The boxplots (panels b,d,f) report the median (black line), upper and lower quartiles (end of the boxes), minimum and maximum (dashed lines) and outliers (circles) of the same parameter sets population represented by the probability distributions (panels a,c,e).

**Figure 3:**
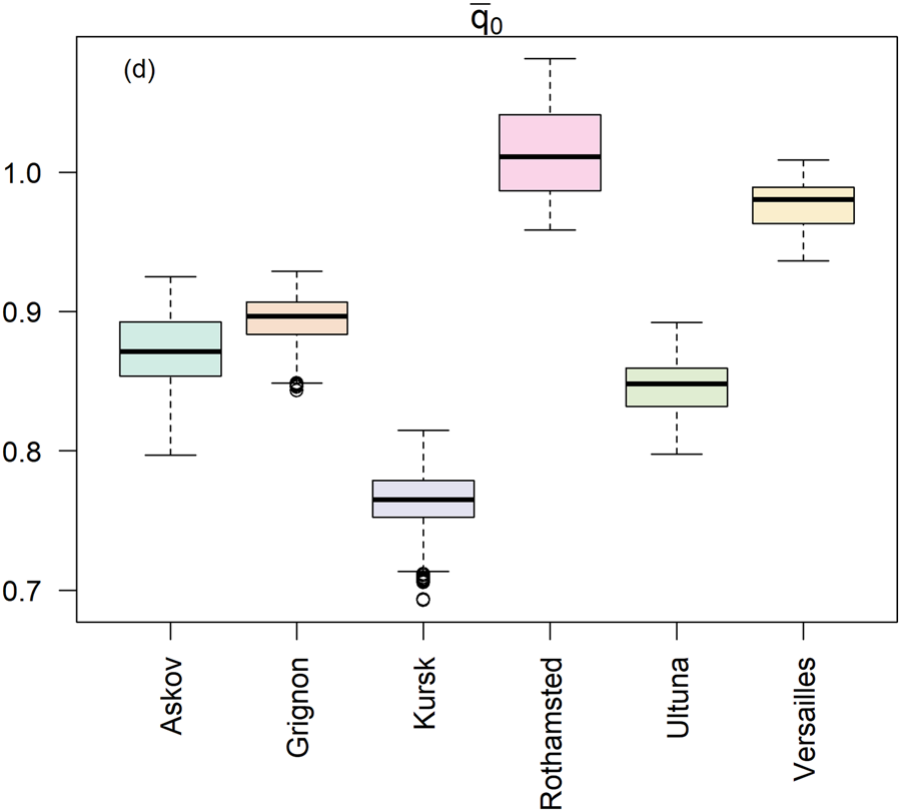
The initial (assuming equilibrium) SOC quality (${\bar{{q}}}_{0}$) at each site according to Eq. 3

The meaning of and calibration approach for each parameter already introduced above are summarized in Table 2. The climatic scaling of *μ*_0_, introduced to consider climatic differences between the sites, was not present in the original model (described in detail in the materials and methods section), and this explains why posteriors for the general kinetics parameters η_11_ and u_0_ differed from previous model formulations. These were however both well-constrained (Fig. 1). The distribution of *q*_0_ values (Fig. 2a) represents SOM quality, which at equilibrium (at the start of the experiment) is correlated only to input quality and quantity. An initial SOM quality distribution skewed toward high qualities, due to high inputs, would therefore be expressed by the model calibration by variations in the *q*_0_ parameter. Former managed (Rothamsted) and unmanaged (Versailles) grassland sites presented the highest average initial SOM quality (${\bar{q}}_{o}$) (Fig. 3), while Grignon, also a former unmanaged grassland, and the two agricultural sites (Ultuna and Askov) presented similar ${\bar{q}}_{o}$. Rothamsted presented significantly higher ${\bar{q}}_{o}$ compared to all other sites, probably as a result of the high organic matter inputs deriving from being a former managed grassland^23^. The lowest ${\bar{q}}_{o}$ was found in the Kursk site, a former grassland managed with a fire suppression regime^24^, possibly reflecting the high content of pyrogenic C at this site^24,25^ and setting this site apart as a unique or special case. Setting Kursk aside, we can group Ultuna, Askov and Grignon as sites with a relatively low initial SOM quality. For the two former sites, this is in line with them being established on former agricultural lands. These three sites presented in a previous study a similar, and relatively low, hydrogen index^26^, proportional to hydrogen to C ratio and linked with compounds generally characterized by lower microbial availability^27^. In general ${\bar{q}}_{o}$ thus seems to be mostly related to the sites’ history.

**Table 2:** Parameters representing autonomous processes in the model, their respective calibration approach and their meaning.

Parameter	Calibration	Meaning
*u*_0_	Generic	Decomposer metabolic rate
*η*_11_	Generic	Rate of decrease in quality
*q*_0_	Local	Initial litter quality
*e*_0_	Local	Decomposer efficiency
*β*_0_	Local*	Edaphic interactions

*The term *β* in the model is calculated according to Eq. 6 combining the calibrated *β*_0_ with a term calculated for each site according to local texture.

The other two local terms, microbial efficiency *e*_0_ (Fig. 2b) and the edaphic term *β*_0_ (Fig. 2c) did not really present any significant difference across all the sites. This result is surprising considering that the soils considered include even the rather peculiar Kursk site for soil type and processes (Chernozem) and environment (former steppe).

### Mechanisms of SOC persistence: ecologically variable or generically valid?

#### SOC persistence initially depends strongly on local SOM quality but later converges to a generic constant

For the local calibrations, the model was most sensitive to *q*_0_ and *β*, while other parameters did not seem to have much impact on local model fits (Appendix 1). Not surprisingly, the model performed better (as measured by the average of the RMSE of each parameter set of the whole Markov chain) when applying a local rather than a general parameterization. This was true in all cases when using the mode of the RMSE distribution (Appendix 2a), and in all cases except Askov, using the mean of the RMSE distribution (Appendix 2b). The latter seems to be caused by an increase in parameter uncertainty, which in the case of Askov becomes crucial due to the relatively short duration of this experiment. With almost no inputs interfering with the SOM quality, initial conditions become crucial and local parameters, in particular *q*_0_, determine the first phase of decomposition dynamics.

Views on SOC persistence have been recurrently challenged in the past^28^, and criticism of the idea of chemical recalcitrance being the main decomposition driving factor is not new^29^. In more recent years, the presence of inherently “recalcitrant“, humified substances has been heavily questioned in favor of more complex protection mechanism involving microbial ecology^6,30^, among other factors. However, our results suggest that one of the main drivers of SOC kinetics over decadal time scales is still, at the epiphenomenon scale, organic matter chemistry^31^. Even if secondary stabilization processes are not necessarily dependent on substrate chemistry itself^32^, still the decomposition of C that forms the more labile SOC pools can be described based on its chemical composition^33^. In our case, this is determined by the land use history of each site (in particular the most recent years before the start of the experiment). The younger SOC, decomposing during the first decade after the start of the experiments (corresponding to the fast pool in discrete compartmental models), is affected the most by local site management, plant community and climate.

**Figure 4:**
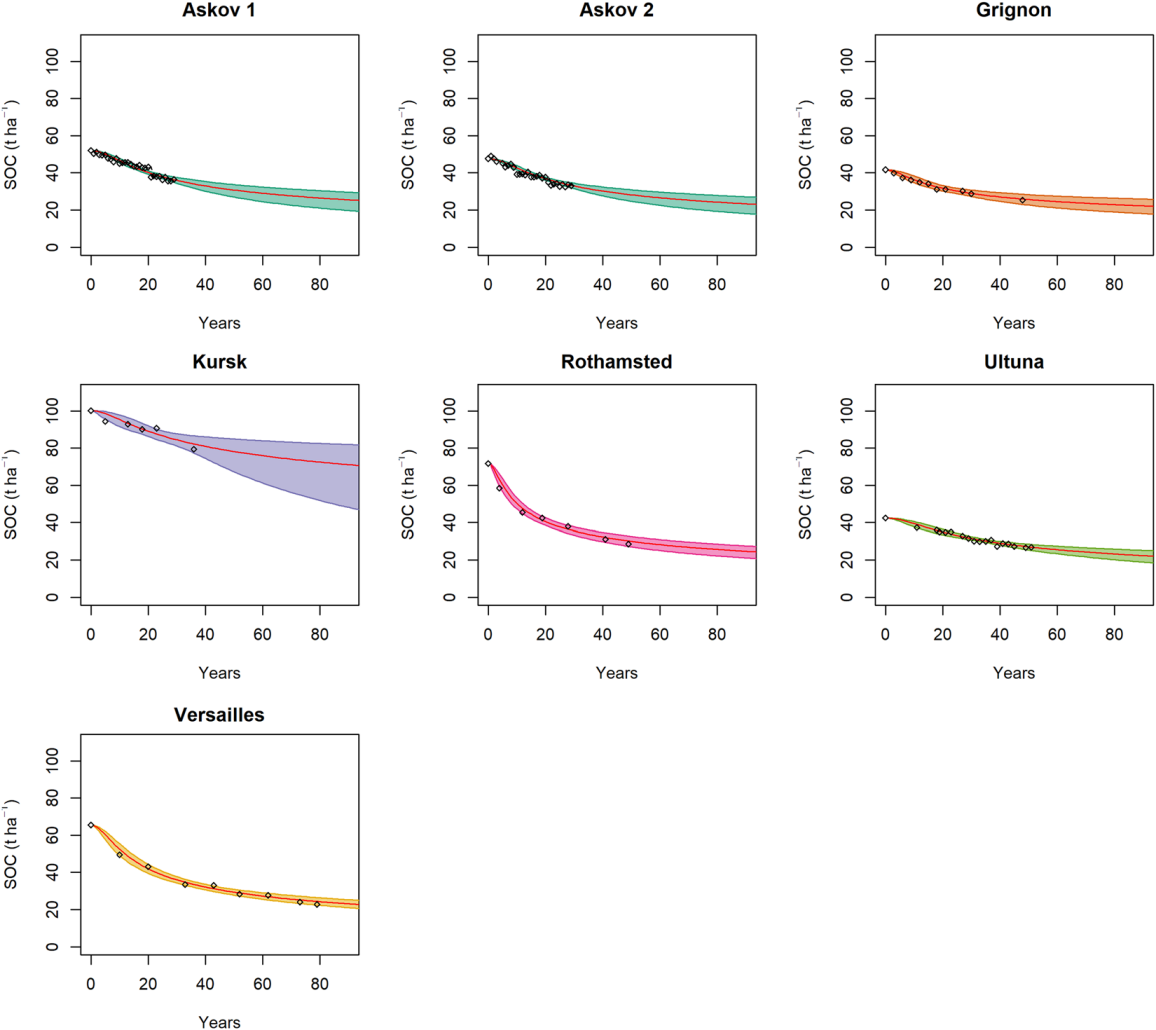
The evolution of SOC in the sites (black empty rombs) and the predictions of the model (colored lines and areas). The continuous red line represents the projection from the best parameter set in terms of RMSE, while the colored areas represent the parameter sets within the 95% quantiles of the RMSE distribution.

The differences reported by the model calibration in the posteriors for ${\bar{q}}_{o}$ (Fig. 3) clearly relate to the history of the sites. However, these differences tend to dissipate over time. After 1–2 decades the initial differences in SOM quality tend to converge to a common value at all sites (Appendix 3). Our model simulates these dynamics using the same value for *η*_11_ and *u*_0_ parameters in all sites (Fig. 4). So while the quality of initial litter inputs has an important role in explaining the initial observed variation between the sites, over the long term, the processes governing SOC decay and its temporal variation in quality were well simulated with general parameter values.

**Figure 5:**
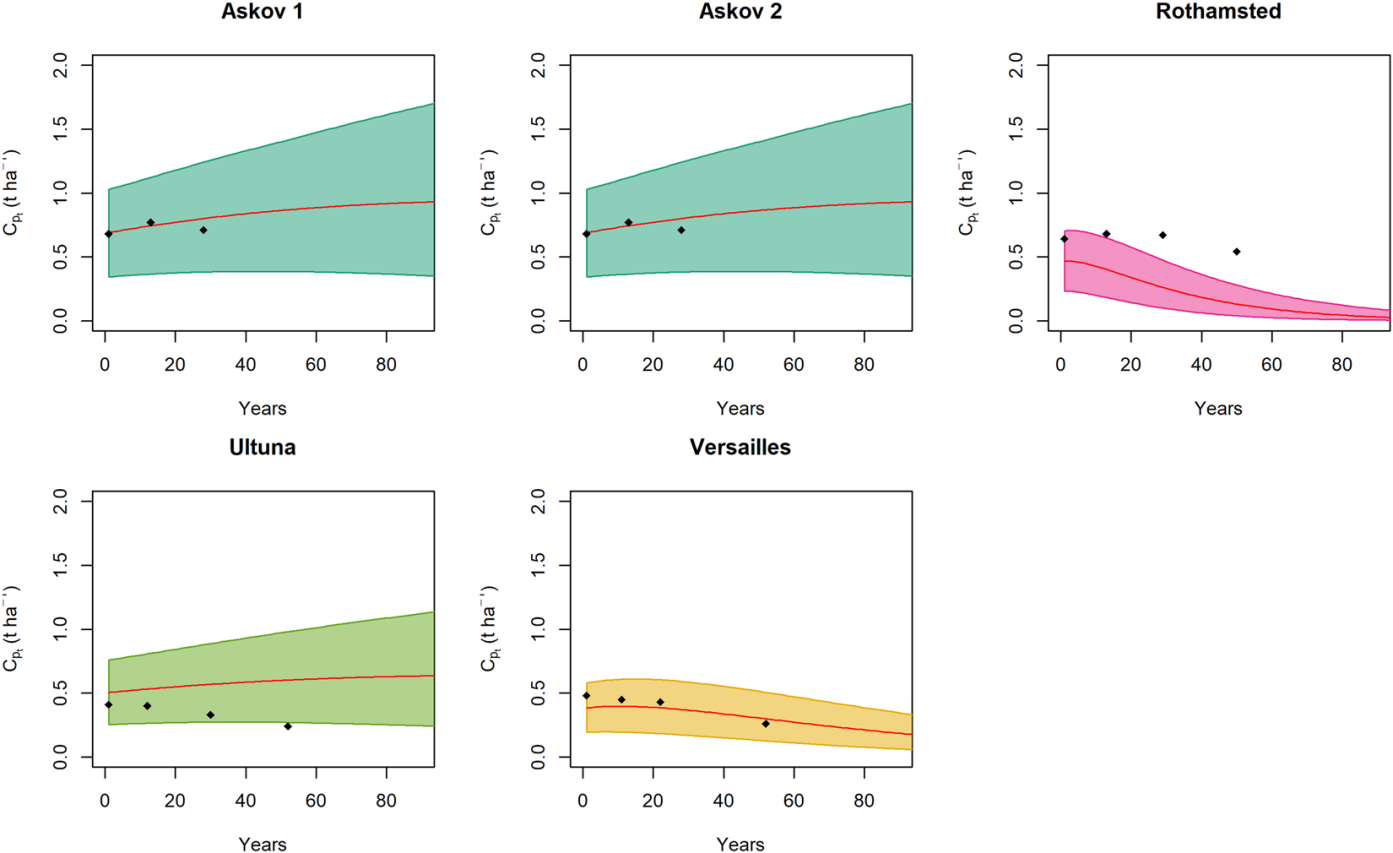
The amount plant material remaining measured (blacks solid rombs) and simulated by the model and described by Eq. 5 (colored lines and areas). The continuous red line represents the projection from the best parameter set in terms of RMSE, while the colored areas represent the parameter sets within the 95% quantiles of the RMSE distribution. No data were measured for Kursk and Grignon.

Wickings *et al*.^34^ identified three possible hypotheses to describe the processes behind SOM decomposition: (*i) the chemical convergence hypothesis* states that organic matter chemistry converges because of molecules cycled inside the microbial biomass, (*ii) the substrate chemistry hypothesis* states that organic matter chemistry evolves because of the selective degradation of different compounds, and (*iii*) the *decomposer control hypothesis* is in fact a modification of hypothesis *i*, and states that differences in microbial communities could eventually cause divergence in SOM chemistry. Barré *et al*.^31^ found that plant-derived lignin compounds decreased over time at LTBF sites (lignin compounds having been fully decomposed after five decades at Versailles, Ultuna and Rothamsted) with a relative enrichment in microbial derived compounds, as well as enrichment in plant-derived alkanes. Their study experimentally demonstrated the shift in SOM quality over time, which was found to be partially due to an enrichment in microbial compounds (driving chemical convergence) but also to selective preservation of some original plant-derived compound even after several decades, demonstrating that degradation is a combination of hypotheses *i* and *ii*. Results confirming hypothesis *i* have been recently reported also by Kallenbach *et al*.^35^. The convergence in chemical composition of SOC has been ascribed to litter material being processed through microbial recycling^36^ Both hypotheses *i* and *ii* and relative experimental results seems to be well represented in the Q model, which simulated the quality convergence (Appendix 3). On the other hand, the model could also represent the persistence of a fraction of the original material (Fig. 5) with the exception of Rothamsted (where the model predicted a higher loss in plant originated compounds than what has been measured). Some discrepancies are to be expected since, compared to what was measured by Barré *et al*.^31^, our definition of quality is based on observed kinetics rather than observed chemistry. The increase in plant-derived alkanes and decrease in plant-derived lignin compounds is represented in the model as a decrease in the quality of the original plant material, but some cases differ from a kinetic determination of quality. Older SOC is considered also to be less energy dense than younger SOC^26^ and to present higher activation energy^37^. The SOM quality convergence predicted by the model (Table 3) is in agreement with this theory since it implies also a convergence of SOC towards higher activation energy^38^, and therefore a convergence of the SOC temperature sensitivity. Our study suggests that, over long time scales, dynamics of SOM decay (considering only the decay of a given starting material) converge toward the same rate of decomposition, which can be described by first-order kinetics and that could represent a consistent unifying principle in soil ecology as envisaged by Fierer *et al*.^39^. The mode of the RMSE (over the whole MC) of the calibrated model was approximately 0.5 t ha^−1^ for all sites, in all cases well below 2.5% of the final C stock values. The persistence of older, more stable, SOC was represented well by general first-order kinetics.

**Table 3:** model predictions of SOC stocks (in t ha^−1^), parenthesis contains minimum and maximum of the 95% C.I.

	Askov 1	Askov 2	Grignon	Kursk	Rothamsted	Ultuna	Versailles
300 years	17.1 (12.1–21.6)	15.7 (11.1–19.8)	16.6 (10.3–20.6)	58.5 (28.7–71.1)	16.6 (14–19.8)	14.9 (12–18.2)	14.1 (12–16.7)
1000 years	12 (6.9–17.2)	11 (6.4–15.7)	11.8 (9.7–14.1)	66.2 (57.8–72.4)	12.2 (10.9–13.5)	11.2 (8.4–13.7)	9.3 (8.3–10.1)
3000 years	7.7 (4.7–12.6)	7 (4.3–11.5)	9.4 (6.3–13.1)	45.8 (34.8–60.8)	13.5 (11.6–15.2)	8.4 (6.1–11.6)	10.8 (9.6–12.4)

#### The dependence of SOC persistence on microbial efficiency is much less important than its dependence on SOM quality

The soil microbial community is ultimately the main driver of SOC decomposition^40^, and, although usually considered functionally redundant and proportional to SOC^20^, its efficiency can vary over a wide range^41,42^. Microbial efficiency expresses the amount of C routed to assimilatory pathways and to microbial cells^41^ and can therefore affect the speed of convergence of SOC chemistry and stabilization. Efficiency also influences the amount of C retained in the soil at steady state.

Our model calibration suggests that eventual minor differences in microbial efficiency between the LTBFs, related to different community structure or metabolic states of the microbial biomass, were not detectable (Fig. 2c,d).The calibrated values were not different between different sites and were well within the range of variation reported for SOC^42,43^.

Chernozem soils tend to be rich in SOC, which is reflected in Kursk having by far the highest SOC stocks among the LTBF sites. While the C:N ratios of Grignon, Rothamsted, Ultuna, Askov and Versailles are 8.44^37^, 8.75^44^, 9.35, 10.27 and 10.15^37^, respectively, the value for Kursk of 13.5^45^ was clearly higher. The current established theory about soil microbial efficiency relates a decreased efficiency with higher C:N ratio for stoichiometric reasons^41^ and would therefore suggest some difference in efficiency particularly in this site, but this was not the case in our model calibration. As revealed also by the very low sensitivity of our model to microbial efficiency compared to *q*_0_ and *β*, the efficiency term did not seem to be needed to explain the observed variation in the LTBF sites.

In general, non-constant metabolic parameters may be required to explain some of the local variability, attributable to hypothesis *iii*^34^. Still from this study results that the variability that remains unexplained by general first-order kinetics is small. Thus, conventional SOC decay models seem in general to be fully capable to describe the dynamics of old and stable SOC. We believe that this conclusion would apply also to any first-order compartmental SOC model, which would express the variability described by *q*_0_ with the initialization of the proportion between the pools and the variability described by *β* with their edaphic functions. The actual efforts to introduce second-order kinetics^15^ in SOC models might be unnecessary when the requirement is to capture longer-term SOC kinetics.

### Recalcitrance, persistence and “inert” SOM

As expected from its structure, even over very long periods of time (>1000 years), the model does not predict a “stable” or “recalcitrant” pool as sometimes conceptualized in the literature^19,46,47^, but rather a continuous decrease of the entire SOC pool. Still, such a decrease slows down with time (Table 3). The further in time we push the assumption of a “stable” SOM the bigger the error we commit, and already after 1000 years the predicted C stocks would already be much smaller (except for Kursk) than the assumed “inert” pool. SOC stocks would even approach 3–5% of initial value in a few millennia (Table 3). Values after three centuries would be already lower, in most cases, than what was formerly predicted as stable pool based on a single exponential function^19^. Considering a model like RothC, that assumes approximately 5% of the total SOC as inert, we can see how already after 1–3 millennia this assumption leads to a different estimation of C stocks.

## Conclusions

Not assuming an inert pool, as done in some models, is crucial for predictions on scales above centuries when the system is losing C. We found that most of the observed differences between the sites are concentrated in the initial decomposition phase, and can be explained with differences in the initial quality of SOM. Over long time scales, the model predicts that the dynamic of SOC will converge at all sites toward the same rate of change, indicating that SOC persistence could follow mechanisms that are common for all sites. Together with recent advances in understanding the chemical composition of SOM and its modifications during the LTBFs experiments, from our results we can deduce that:

- The chemical composition of SOC in the beginning of the experiment appears to be what locally distinguishes initial SOC kinetics. We cannot exclude that together with soil edaphic parameters, soil microbial efficiency (influenced by nutrient stoichiometry) might still play a role in defining SOC kinetics, but this role is marginal in comparison to initial SOC quality and was not detectable in our study.
- Over long time scales and without the influence of new organic matter input, initial quality effects dissipate and the variables describing SOC persistence and kinetics converge. Old SOM has properties that are similar across different environments and soils and that can be described with unified principles.
- The kinetics of SOM decay under conditions of no C inputs seems, on a scale of decades, not influenced by factors other than SOM quality

In general, at least for older C decaying in absence of external inputs, resorting to second order kinetics does not seem necessary at these scales for predicting SOM with high accuracy.

## Methods

### Data used for calibration

The LTBF network is presently composed of six experiments at five sites (Table 1), namely Askov (Denmark, two experiments), Grignon (France), Kursk (Russia), Rothamsted (United Kingdom), Ultuna (Sweden) and Versailles (France), all characterized by long experimental duration ( > 30 years) (Table 1). All sites have been kept free from vegetation and other C inputs (P. Barré *et al*.^19^ and reference therein). Before the start of each experiment the sites had different land uses. The Kursk site, initiated in 1965, is the most unique since it was a steppe (although it was then cultivated for approximately two centuries before the start of the experiment), and it developed as particularly rich in SOM as all Chernozems. These are soils from prairie continental regions, with a mollic horizon on top, where organic matter accumulates because of climatic factors (reduced temperature and waterlogging during winter and dry periods in the summer when temperature would otherwise allow for decomposition) and in general the particular vegetation. Organic matter gets then stabilized by chemico-physical interactions mediated by soil fauna and high Ca content^48^, resulting in a high SOC content with some resistance to decomposition (at least compared to organic soils such as peat). Sampling depth is 25 cm. The Askov site, a sandy Luvisol, was a mixed landscape, with heathland and shrublands with patches of grassland. Sampling depth is 20 cm. Ultuna, initiated in 1956 on a Cambisol, was in agricultural use for centuries before the start of the experiment. Sampling depth is 20 cm. The sites of Grignon, initiated in 1959, and Versailles, initiated in 1928, were both unmanaged grassland. Sampling depth is 25 cm for both, and both sites are on a Luvisol. The site at Rothamsted, initiated in 1959 also on a Luvisol, was a managed grassland. Sampling depth is 23 cm. For more details about the sites, refer to Barré *et al*.^19^. C stocks were calculated on an equivalent soil mass basis according to Barré *et al*.^19^, so the variation in the bulk density (and consequent variation of the initial topsoil depth) observed during the duration of the experiments is considered in the C stocks^33^. Weather data were derived from https://www.ncdc.noaa.gov/cdo-web/.

### The Q model and the concept of SOM quality

The Q model^5^ is a first-order organic matter model based on the *continuous quality* theory^38,49,50^. In this study the term quality is assumed inversely proportional to persistence, and it is represented by the model with a continuous distribution rather than with a set of discrete pools as in most other SOC models^20,21,51^. This continuous distribution is modified to represent the evolution of the substrate over time towards a less decomposable average composition. All systems were assumed to be at steady state before the start of the experiment, and since they have no inputs we could use a simplified version of the core model equation (as presented by Hyvönen, Ågren and Bosatta^52^). This function describes the decay of SOC depending on how the average SOC quality ($\bar{q}$) changes relative to the quality of the input (*q*_0_), when input is stopped:

1 $${C}_{t}={C}_{ss}{(\frac{{\bar{q}}_{t}}{{q}_{0}})}^{[\frac{(1-{e}_{0})}{{\eta }_{11}{e}_{0}}]-\beta }$$

where *C*_*t*_ refers to amount of SOC at time t, while *C*_*ss*_ refers to amount of SOC at steady state (in our case assumed at the beginning of the experiments). For the meaning of the parameters please refer to Table 2. The term $(\frac{{\bar{q}}_{t}}{{q}_{0}})$ expresses the average quality of the SOM at time t (${\bar{q}}_{t}$) relatively to the initial litter quality according to:

2 $${\bar{q}}_{t}=\frac{{q}_{0}}{{(1+\beta {\eta }_{11}{u}_{0}{r}_{e}{q}_{0}^{\beta }t)}^{\frac{1}{\beta }}}$$

For the meaning of the parameters please refer to Table 2. Compared to previous formulations^52^ the term *f*_*c*_ (carbon concentration in the decomposers biomass) has been embedded in *u*_0_, while the term *r*_*e*_ was added for climate normalization (explained in detail below).

The initial litter quality *q*_0_ determines the quality of SOM at equilibrium (${\bar{q}}_{0})$, condition assumed before the start of each experiment, according to:

3 $${\bar{{q}}}_{0}=\frac{1-{{e}}_{0}-{{\eta }}_{11}{{e}}_{0}{\beta }}{1-{{e}}_{0}-{{\eta }}_{11}{{e}}_{0}({\beta }-1)}\cdot {{q}}_{0}$$

From which is clear how the average SOM quality and the quality of the organic input (litter) are linearly proportional. In field conditions and even with the same input quality, a site with higher inputs would end up having slightly higher average SOM quality, because it takes some time for the added material to be processed. Initial litter input quality *q*_0_ is proportional to the average initial SOM quality ${\bar{q}}_{o}$ (Eq. 3), although such proportionality is scaled also by other local parameters (*β* and *e*_0_).

A recent study by Barré *et al*.^31^ offered more specific information on the chemistry of SOM in the time series of the LTBF sites, in particular about the fraction of plant-derived materials remaining over time. We assumed that this plant material (*C*_*p*_) corresponds to the material of quality q_0_ in the model, and this can be calculated during the SOM accumulation phase before the start of the experiment and so in the presence of input *I*:

4 $$\frac{d{C}_{p}}{dt}=I-\frac{{u}_{0}{q}_{0}^{\beta }}{{e}_{o}}{C}_{p}$$

Which for steady state can be written as:

5 $${C}_{{p}_{ss}}=\frac{{e}_{0}}{{u}_{0}{q}_{0}^{\beta }}\,I$$

In case of a bare fallow, which has no inputs, the plant fraction remaining as a function of time is calculated in the model as:

6 $${C}_{{p}_{t}}={C}_{pss}{e}^{-\frac{{e}_{0}}{{u}_{0}{q}_{0}^{\beta }}t}$$

Equation 5 was used in the calibration to relate the measured plant fraction remaining with Eq. 1 on the measured SOC stocks. The two equations were calibrated simultaneously.

### Known independent variables: moisture, temperature, clay effects

All weather effects on SOC decay were calculated based on annual averages of daily measurements. The temperature effects on SOC kinetics are based on Lloyd & Taylor (1994, eq. 8) until a temperature of 35.14 °C. Activation energy for driving the temperature function was taken from the average values measured for the bare fallow sites (Lefevre *et al*.^37^) and was set to 59.46 kJ mol^−1^, so not far from the original value of 53 kJ mol^−1^^9^. Although we know that the activation energy had some minor variation over the years of the experiment^37^ we assumed it constant in order to test the capability of a first order kinetics model to represent the observed data. Using a variable activation energy would have influenced the test by providing a variation of the kinetics not considered by the model. After rescaling, the temperature function generates a temperature reduction function (*re*_*temp*_, Appendix 4 and 5) between 0 and 1. We calculated the soil water balance based on Andrén *et al*. (Eq. 1)^53^ with updated pedotransfer functions^54^. The potential evapotranspiration (PET) was calculated based on the Penman-Monteith function^55^ with aerodynamic resistance from Liu *et al*.^56^, plus a scaling term (0.9) from data from the Rothamsted bare fallow^57^. Actual ET was calculated according to Andrén *et al*.^53^. The soil water saturation fraction was used to drive the moisture function developed by Moyano *et al*.^58^. This function gives a moisture reduction value between 0 and 1 (*r*_*moist*_, Appendix 4 nd 5). The interaction between temperature and moisture was considered multiplicative (*r*_*e*_ = *r*_*moist*_ × *r*_*temp*_). To make possible a comparison between sites situated in different climatic zones, we considered the relative climate driven change in each site and filtered climate effect out, the term *r*_*e*_ was normalized using as reference the average *r*_*e*_ over all the sites (Appendix 2). We assumed that climate would affect SOC decomposition by modifying the metabolism of the decomposer community, so we used *r*_*e*_ to rescale the decomposer metabolic parameter *u*_0_ on an average climate for all the sites. For future predictions we used the average normalized *re*_*clim*_ for each site^21^.

We represented clay effect according to Bosatta & Ågren^59^, as a modifier of *β*:

7 $$\beta ={\beta }_{0}+0.01\cdot \chi $$

where *β*_0_ is a calibrated term and *χ* is the measured relative concentration of clay (%) in the soil.

### Model calibration

The model was calibrated within a Bayesian probability framework by running 4 independent Monte Carlo Markov chains (MCMC) of 300.000 elements within the space of model priors. This means that for each of the elements of the chain a value for each parameter was chosen according to their prior probability distribution by a probabilistic sampler, and the model run with such parameter values. The sampler works, according to the law of large numbers, by comparing the likelihood of each parameter combinations (roughly proportional to the RMSE) with a randomly generated likelihood. The principle is that whenever the likelihood of the parameter set is better than random then that set is kept and the chain (where, we remind, one element is one full parameter set) is updated, otherwise it is refused and the chain keeps the previous value. This allows us to calculate statistics on the whole MCMC, deriving probability distributions and uncertainties according to prior knowledge and data. Convergence and autocorrelation of the chains were checked visually for the most relevant parameters (Appendix 6–8). We also run a Gelman-Rubin diagnostic test (Appendix 9) measuring the convergence of the four independent chains. Values < 1.1 means conventionally good mixing although the metric is just a rough estimate^60^ and varies a lot depending on the correlation between parameters (more correlated parameters, or in other words an overparameterized model, mean a more difficult space to explore and a consequent lower Gelman-Rubin diagnostic value). Data on the proportion of plant C in total SOC^31^ are unpublished data shared by one co-author. C stocks were calculated on an equivalent soil mass basis according to Barré *et al*.^19^. The SOC data, originally irregular time series derived from Barré *et al*.^19^, were homogenized to regular time series by linearly interpolating the data to annual resolution. The SOC model was calibrated with a Metropolis-Hastings sampler, written in JAGS^61^. Errors were expressed as Gaussian functions with an error parameter corresponding to the average standard deviation of each time series for each site. We calibrated by running four separate chains, each of 100 000 iterations and with 10 000 iterations excluded as burn-in time. All analyses have been performed with R^62^.

### Potentially unknown independent variables: choice of local parameters

We first calibrated the model with the same parameter values for all sites, using the 100 000 iterations to perform a Hornberger-Spear-Young (HSY) sensitivity analysis^63^. We defined two bins of *behavioral* and *non-behavioral* parameter sets with a threshold (specific to each site) corresponding to the 5% quantile of the RMSE of all the parameter sets in the Markov chain. All parameters sets with an RMSE below this threshold were considered behavioral. We then summarized the probability distribution distance between them with the Kolmogorov-Smirnov (KS) distance for each site, which gives a rough estimation of the sensitivity of the model to each parameter in each site (Appendix 1). On average among all sites, the highest sensitivity by far was found for *q*_0_, (average KS distance of 0.180). The second ranking parameter was *β* (average KS distance of 0.165), while the model was much less sensitive to any of the other parameters (with an average KS distance of 0.051 for *e*_0_, 0.064 for *u*_0_ and *η*_11_,), We therefore decided to calibrate *q*_0_ locally (based also on measured data^26,31^), and to add to *β* (calculated according to Eq. 6) a local term *β*_0_. Since recent studies are pointing out that microbial efficiency might be another important variable to consider^41^, we decided to include *e*_0_ in the local calibration. The microbial metabolic parameter *u*_0_ was considered to be generic based on the idea that, while efficiency of microbes is influenced by stoichiometric nutrient constraints^13^, the dynamics of microbial communities in soil is a direct consequence of metabolism which follows thermodynamic laws^64,65^. Kinetic parameters in compartmental models are usually assumed as generic^52^ as in most SOC models^20,21^. SOC decay is also dependent on microbial metabolism and we expect it to follow universally valid thermodynamic laws^9,66^. Some small local variations in the SOC decay kinetics could eventually be caused by priming, but the priming effect in a bare fallow is supposed to be negligible because of the lack of inputs. We therefore assumed *η*_11_ to be a generic parameter. We then run a second calibration with the parameter calibration strategy summarized in Table 2.

### Model priors

We took priors for the model parameters whenever possible from previous studies. We selected relatively undefined and wide prior probability distributions for the generic parameters, *u*_0_ and *η*_11_, since these two parameters are the most difficult to derive from measurements and are therefore uncertain. Both *u*_0_ and *η*_11_ relate to kinetic terms of SOC decay, are latent variables, and to some extent interact. They have usually been set as fixed in previous studies (e.g. Hyvönen *et al*., 1998^52^). Because of such uncertainty, for *η*_11_ and *u*_0_ we used uniform priors within a range of ±100% of the literature value (0.36 for *η*_11_^52^ and 0.5 × 0.98 for *u*_0_, which are the literature values for *f*_*c*_ and *u*_0_, respectively^52^.

For the local parameter *β*_0_, which has been treated more specifically in the literature^59^, we used a normal distribution with mean 0.7 and coefficient of variation of 10% (but truncated within a range of ±33% of the literature value). For the other two local parameters, *q*_0_ (litter input quality distribution) and *e*_0_ (efficiency of decomposers), we also used Gaussian priors with the same coefficient of variations of 10%. For *q*_0_, the distribution was centered at 1.08, truncated prudentially between 0.5 and 1.5, which is a bit wider than previously reported in the literature^49,67^. For *e*_0_ the distribution was centered at 0.3 and was truncated between 0 and 0.6 according to Sinsabaugh *et al*.^41^. The average microbial efficiency, *e*_0_ in the Q model but commonly referred to as carbon use efficiency (CUE), is a crucial parameter defining how much of the C is respired and how much is instead recycled and incorporated into SOM. It can therefore be roughly considered proportional to the humification ratio”^33^, although on a different scale, and it strongly influences SOC kinetics. The model we used, working in annual steps, neglects variations of efficiency (such as seasonal fluctuations, Tucker *et al*. 2013), so that our analysis applies a single efficiency value to each site representing the average over the whole experimental period.

## Supplementary information

Supplementary material

## Data Availability

All data from the LTBF network can be requested to the data holders (mentioned in Barré *et al*.; 2010, and in the acknowledgement of this manuscript).
