# Supplementary material for: Generic parameters of first-order kinetics accurately describe soil organic matter decay in bare fallow soils over a wide edaphic and climatic range
Source: Sci Rep. 2019 Dec 30;9:20319. doi: 10.1038/s41598-019-55058-1 (PMC6937324; doi:10.1038/s41598-019-55058-1)
Supplement: Supplementary file 1 — Supplementary material [file 41598_2019_55058_MOESM1_ESM.pdf]

Generic parameters of first-order kinetics accurately describe soil organic matter decay in bare fallow soils over a wide edaphic and climatic range

Supplementary material

Authors: Lorenzo Menichetti[1]*, Göran I.Ågren[1], Pierre Barré[2], Fernando Moyano[3], Thomas Kätterer[1]

[1] Department of Ecology, Swedish University of Agricultural Sciences (SLU), Box 7044, 75007 Uppsala, Sweden

[2] Laboratoire de Geólogie de l'ENS, PSL Research University – CNRS UMR8538, 75005 Paris, France

[3] Georg-August Universität Göttingen, Büsgenweg 2, 37077 Göttingen, Germany

*Lorenzo.Menichetti@slu.se

	Askov1	Askov2	Grignon	Kursk	Rothamsted	Ultuna	Versailles	Average
q_o	0.107	0.074	0.256	0.381	0.284	0.071	0.091	0.18
u	0.072	0.062	0.059	0.051	0.067	0.075	0.059	0.064
β	0.09	0.08	0.273	0.347	0.244	0.053	0.065	0.165
η_{11}	0.05	0.09	0.043	0.07	0.05	0.043	0.102	0.064
e_o	0.045	0.044	0.058	0.061	0.049	0.038	0.068	0.052

Appendix 1: the Kolmogorov-Smirnov distance between behavioral *and* non-behavioral *parameter sets for each parameter and site*

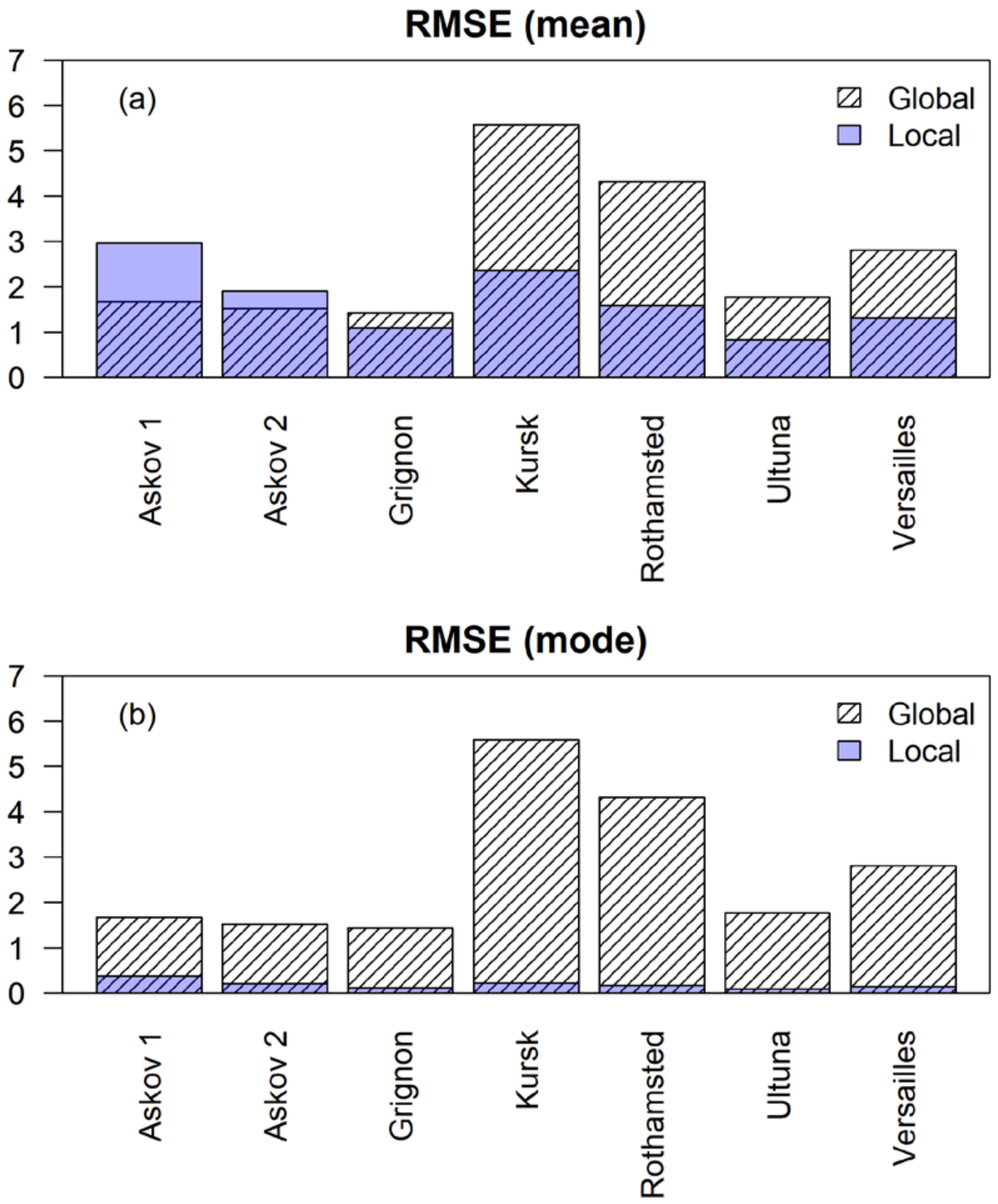

Appendix 2: the mean (a) and mode (b) of the RMSE of the initial calibration (generic) with all parameters assumed to have the same value for each site, and the calibration with the three local parameters (q_0, e_0, [illegible]$_0$) *for each site.*

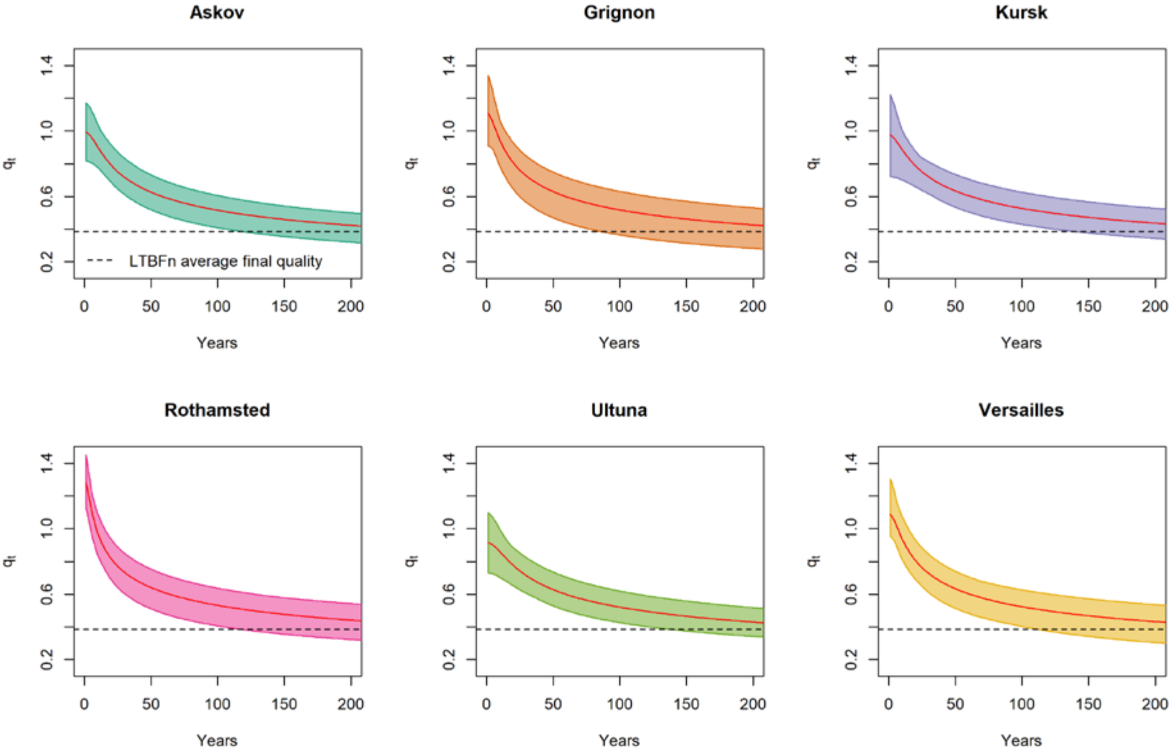

Appendix3: the evolution of quality of SOC over time predicted by the model for the six sites (the dashed line represents the average quality after 200 years). The continuous red line represents the projection from the best parameter set in terms of RMSE, while the coloured areas represent the parameter sets within the 95% quantiles of the RMSE distribution. The Askov site is here represented as one single site since no measurements are involved and the parameters generating the plots are unique to each site.

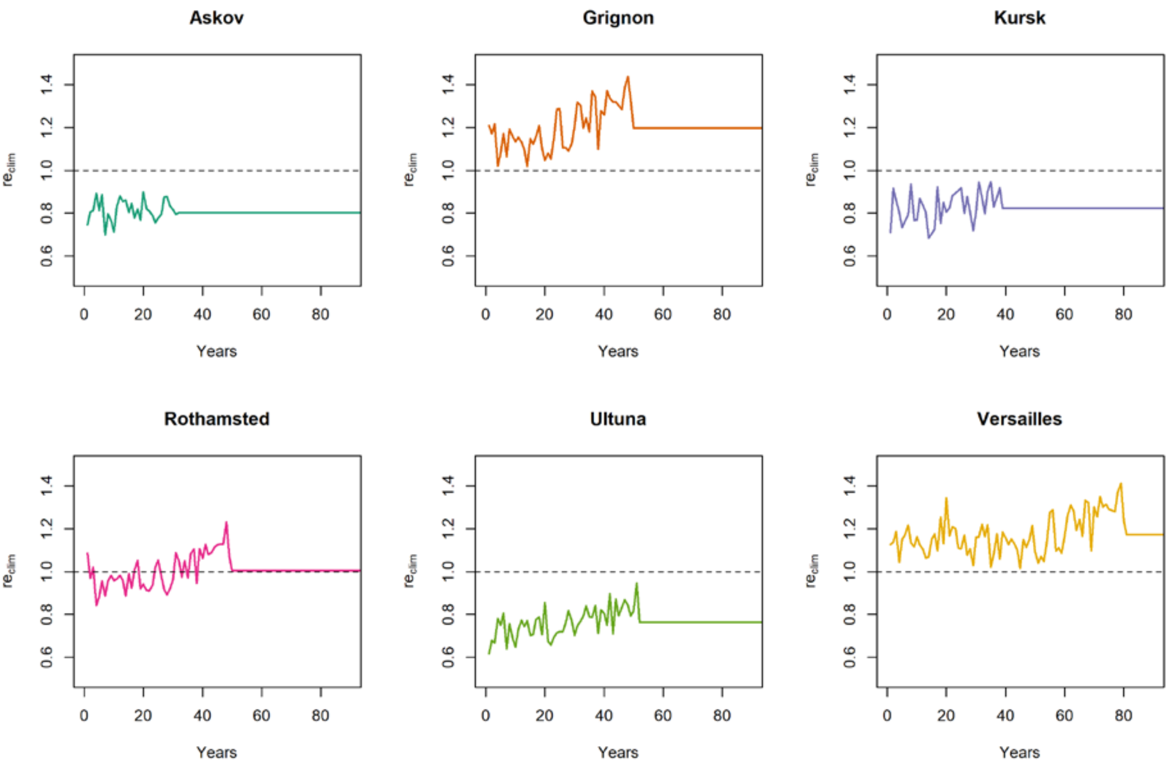

Appendix 4: the final normalized climatic reduction factors for each site, combining together soil moisture and temperature effects. The represented oscillations are proportional to precipitation and temperature time series in each sites.

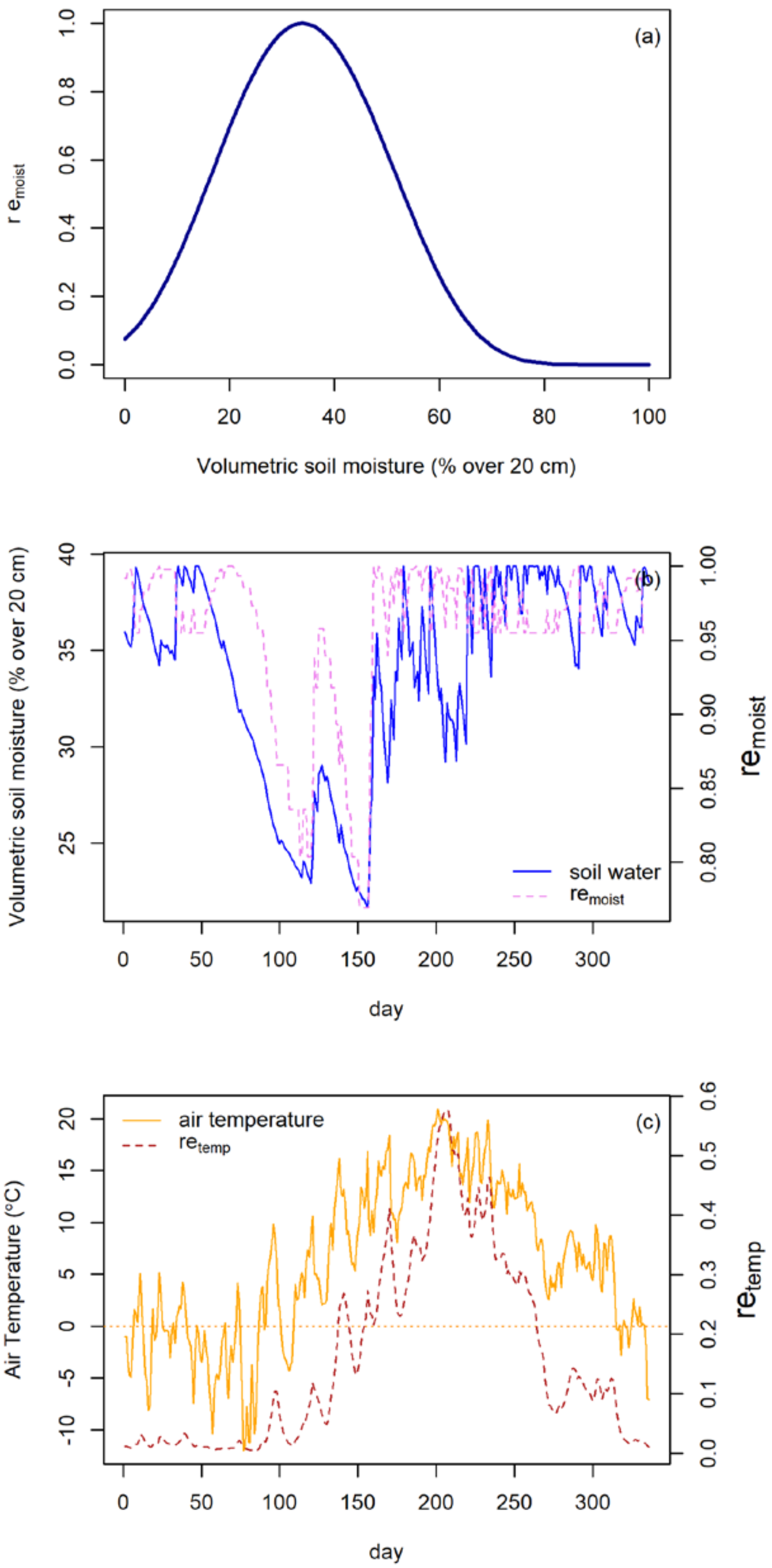

Appendix 5: example of the calculation of the (non-normalized) reclim factor, here for the Ultuna site in year 1th (time is expressed in days from the start of the experiment). Panel a) depicts the water reduction function, panel b) the moisture reduction factor with the main driving variable and panel c) the temperature reduction factor with the main driving variable

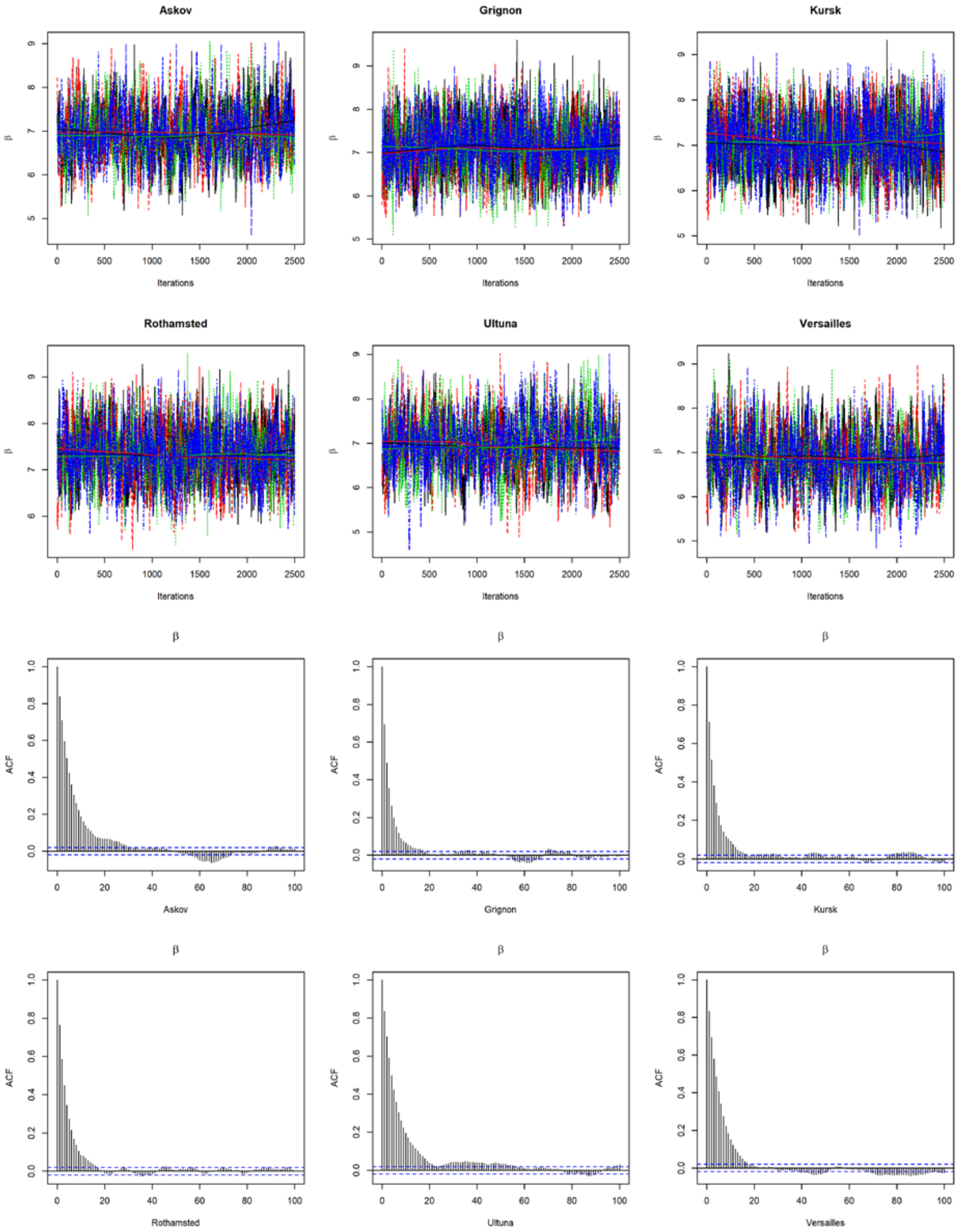

Appendix 6: the trace of the four independent Markov chains and their autocorrelation for the six sites (parameter β)

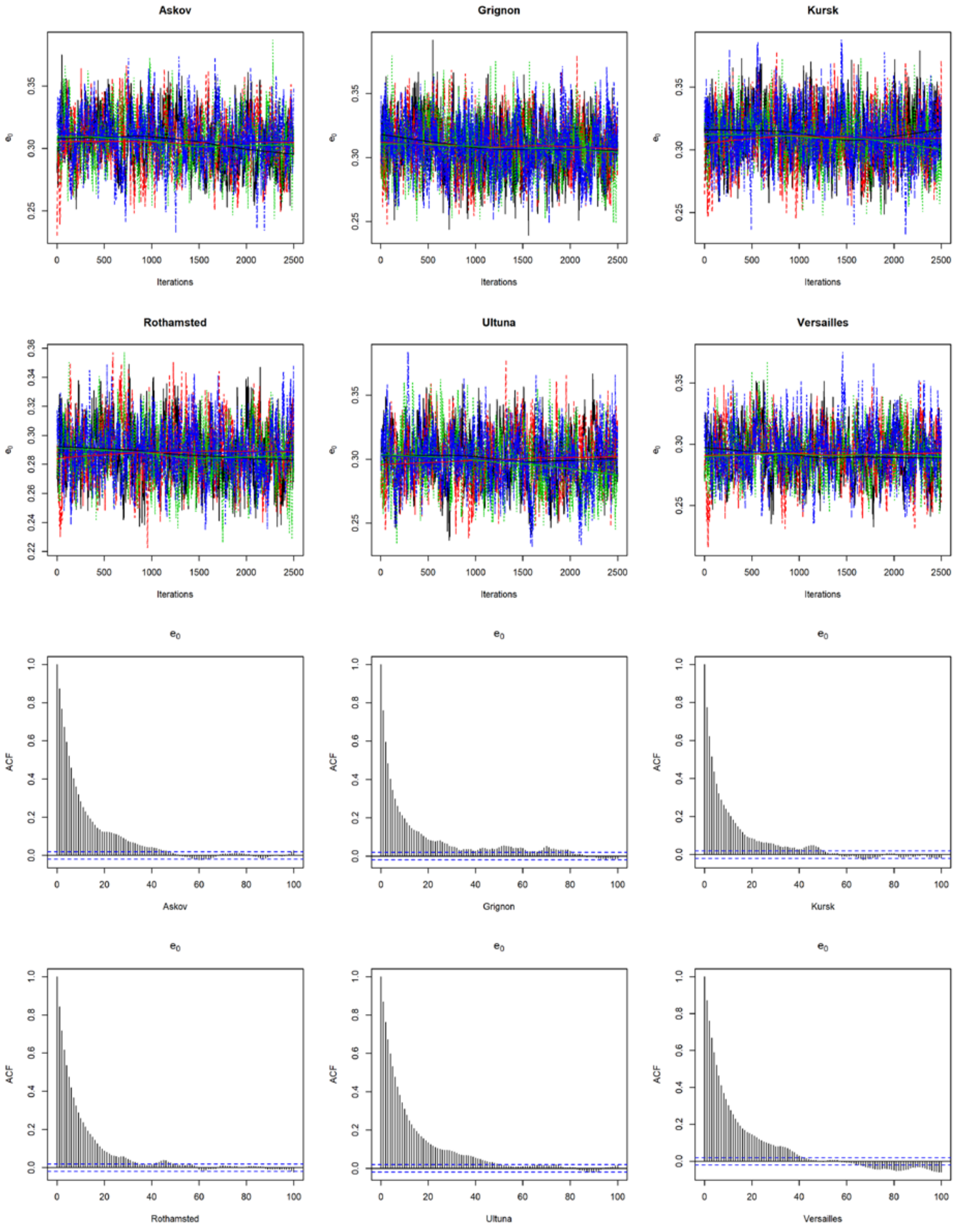

Appendix 7: the trace of the four independent Markov chains and their autocorrelation for the six sites (parameter e_0)

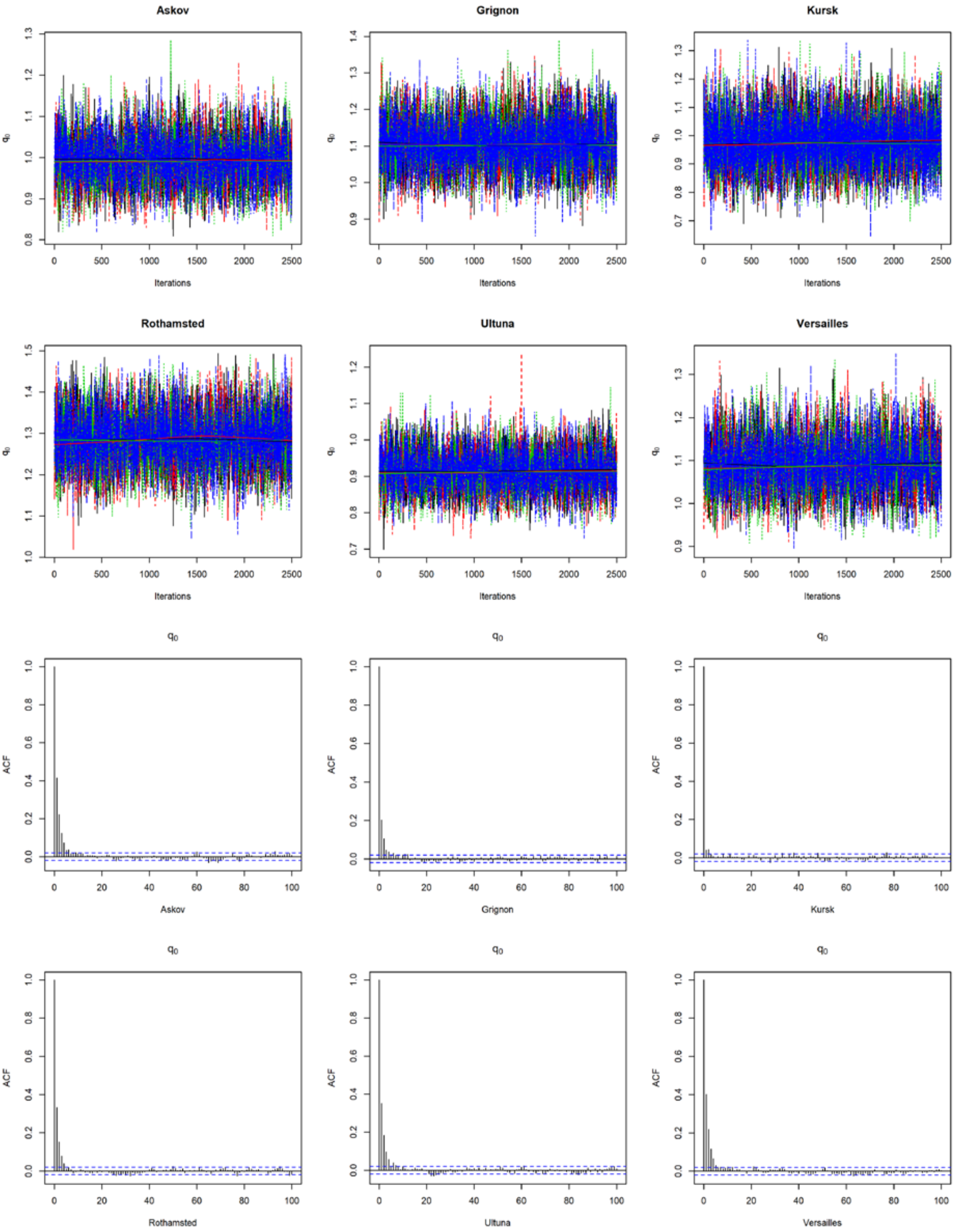

Appendix 8: the trace of the four independent Markov chains and their autocorrelation for the six sites (parameter q_0)

	Askov	Grignon	Kursk	Rothamsted	Ultuna	Versailles
Point estimate β	1.00	1.00	1.00	1.00	1.02	1.01
Upper C.I. β	1.01	1.01	1.01	1.01	1.05	1.02
Point estimate e_0	1.01	1.01	1.00	1.02	1.02	1.01
Upper C.I. e_0	1.02	1.01	1.01	1.05	1.05	1.04
Point estimate q_0	1.00	1.00	1.00	1.00	1.00	1.00
Upper C.I. q_0	1.01	1.00	1.00	1.01	1.01	1.00

Appendix 9: Gelman-Rubin convergence of the chains for the most relevant parameters (values <1.1 means conventionally good convergence [60])
